# A comparison of the diagnostic ability of large language models in challenging clinical cases

**DOI:** 10.3389/frai.2024.1379297

**Published:** 2024-08-05

**Authors:** Maria Palwasha Khan, Eoin Daniel O’Sullivan

**Affiliations:** ^1^Kidney Health Service, Metro North Hospital and Health Service, Brisbane, QLD, Australia; ^2^Institute of Molecular Bioscience, University of Queensland, St Lucia, QLD, Australia

**Keywords:** artificial intelligence, machine learning, clinical medicine, LLM, diagnostics

## Abstract

**Introduction:**

The rise of accessible, consumer facing large language models (LLM) provides an opportunity for immediate diagnostic support for clinicians.

**Objectives:**

To compare the different performance characteristics of common LLMS utility in solving complex clinical cases and assess the utility of a novel tool to grade LLM output.

**Methods:**

Using a newly developed rubric to assess the models’ diagnostic utility, we measured to models’ ability to answer cases according to accuracy, readability, clinical interpretability, and an assessment of safety. Here we present a comparative analysis of three LLM models—Bing, Chat GPT, and Gemini—across a diverse set of clinical cases as presented in the New England Journal of Medicines case series.

**Results:**

Our results suggest that models performed differently when presented with identical clinical information, with Gemini performing best. Our grading tool had low interobserver variability and proved a reliable tool to grade LLM clinical output.

**Conclusion:**

This research underscores the variation in model performance in clinical scenarios and highlights the importance of considering diagnostic model performance in diverse clinical scenarios prior to deployment. Furthermore, we provide a new tool to assess LLM output.

## 1 Introduction

Accurate diagnosis is a fundamental step in high quality clinical care. The potential for large language models (LLMs) to provide clinical support and to improve diagnostic abilities of clinicians is increasingly appreciated and the subject of much research interest (Cascella et al., 2023; Chen et al., 2023; Chirino et al., 2023; Huang et al., 2023; Kleesiek et al., 2023). While it is becoming apparent that publicly available LLMs can produce impressive results in clinical vignettes, it is not known which model is most currently most useful to a working clinician, nor is there a reproducible way to compare LLMS output (Kung et al., 2023).

Here, we propose a simple, rapidly deployed and actionable grading rubric to compare the clinical utility of the output of LLMs. We use this rubric to grade the output of three publicly available models, ChatGPT (GPT3.5, April 2024 OpenAI, San Francisco, United States), Bing (GPT4, April 2024 Microsoft, Redmond, United States) and Gemini (Pathways Language Model -PaLM v1.5, April 2024, Google, Mountain View, USA) when asked provide clinically appropriate diagnosis and differential to a clinical vignette. To simulate challenging cases where a competent clinician may realistically have need of diagnostic support, we selected a range of clinical cases presented in the New England Journal of Medicines (NEJM) Cases series. These cases have sufficient complexity to move beyond the shorter, “classic” vignettes of simple medical examination and provide challenge to a post graduate clinician. To measure the ability of the models to provide clinically useful support, we designed a simple rubric to grade output. This focused on both the ability to provide an accurate and understandable diagnosis, appropriate differentials as well as providing clinically safe output, free of hallucinations or dangerous suggestions and presented in a readable, understandable manner.

## 2 Methods

Ten distinct clinical cases were selected from clinical cases in the NEJM case series, covering a spectrum of medical conditions, from ovarian masses to toxic shock. The case discussions in the NEJM are presented initially as a vignette of the history of the presenting complaint, relevant initial investigations, and examination findings. Thereafter, an expert discussion follows, explaining the clinical reasoning behind relevant differentials. This initial vignette was the input provided to models. Specifically, the three models under investigation, Bing, ChatGPT4 and Gemini were asked to “please provide a diagnosis and relevant differential diagnosis to the following case,” and the unedited text (and data tables where relevant) was provided. The free, consumer facing versions of the models were used, as open to the general public and accessed via the respective websites. No other programs or plugins were used. In this manner, models were prompted using identical text input. The specific cases chosen were: Ruptured ovarian cyst, Systemic Lupus Erythematosus, Insulinoma, Posterior Reversable Encephalopathy Syndrome, Renal Cryptococcosis, Necrotizing Anterior Scleritis, B12 deficiency, HNF1b mutation, SARS-ARDS and Septic shock syndrome.

**Figure 1:**
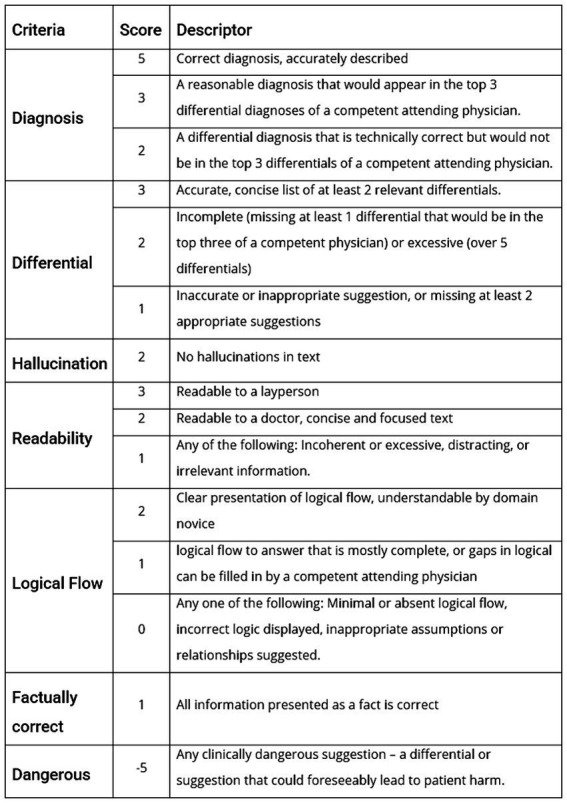
The grading rubric to assess LLM output when presented with the case vignette.

A Rubric was created to grade the output of the models based on real world clinical interpretability and utility. The correct diagnosis and relevant differentials are provided by the NEJM in the case, so this was used as the ground truth for evaluation of accuracy. However, other metrics such as readability and potentially dangerous suggestion as subject by nature, so multiple medical clinicians from a range of specialties, countries and levels of seniority provided scores to allow generalisability. The grading rubric developed is presented in Figure 1.

The performance metrics the rubric focused on were correct diagnosis, differential diagnosis, hallucination, readability, explainability, incorrect statements, and overall subjective assessment of potentially risk suggestions. The grading rubric was applied independently by 10 physicians from 3 different countries and a range of seniorities and specialties, and an average score calculated.

Scores were compared between models using ANOVA test and Tukey’s *post hoc* testing. *p*-values below 0.05 were considered statistically significant. Analysis performed in R version 4.3.2. Inter-rater reliability was assessed using the intraclass correlation coefficient (ICC) to measure the consistency of scores assigned by multiple raters. A two-way consistency model (ICC type: consistency) was applied to evaluate agreement among raters. The ICC was computed based on the average scores provided by each rater.

Statistical analysis was performed using the icc() function from the irr package in R. The ICC value, along with its 95% confidence interval (CI), was calculated to quantify the level of agreement among raters. A significance test (F-test) was conducted to determine whether the ICC significantly deviated from zero, indicating reliable agreement among raters.

## 3 Results

**Figure 2:**
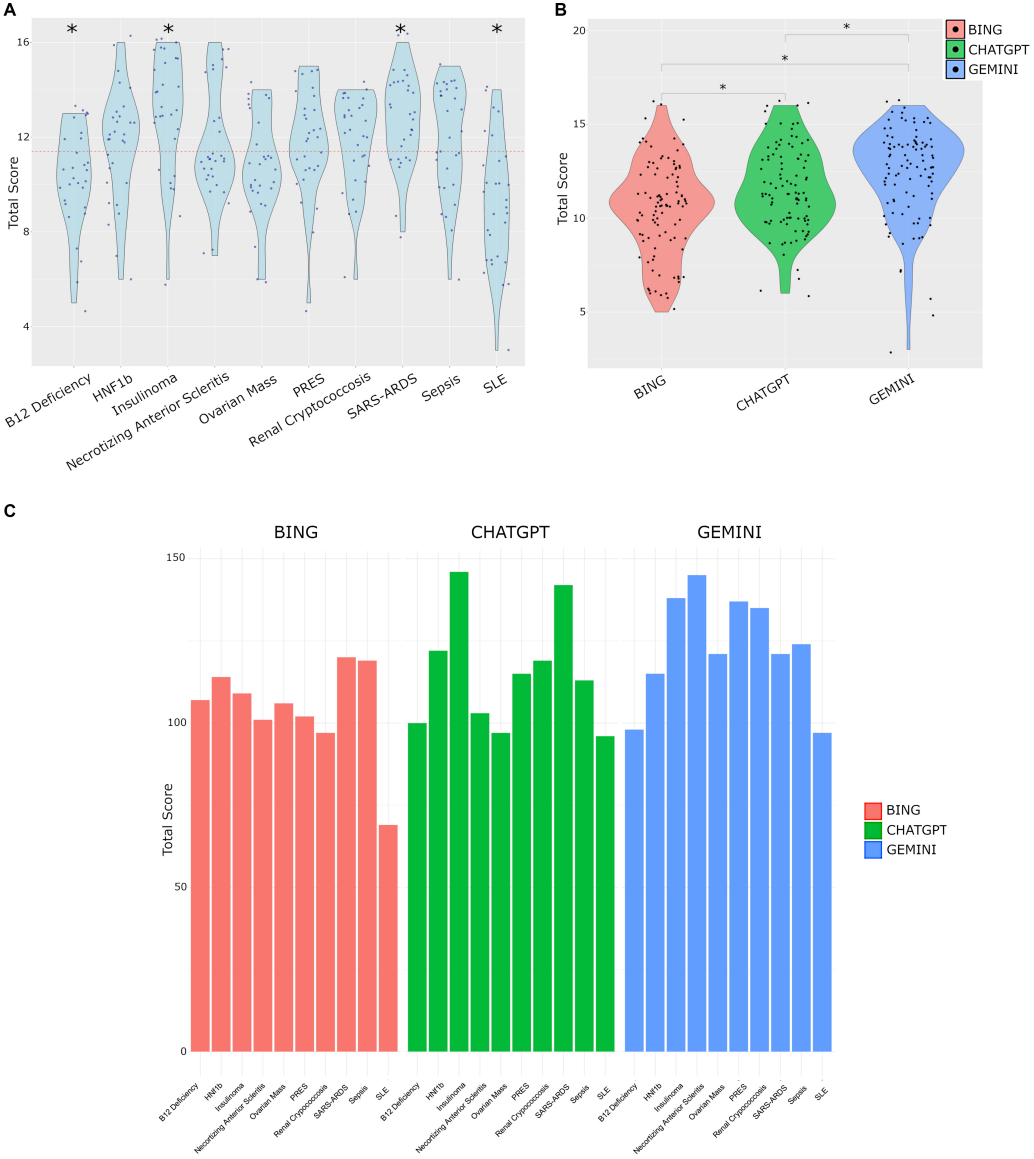
**(A)** Total scores across all cases. Red dotted line represents the mean score (11.4). Significant differences tested by *T*-test vs. mean score, *p* < 0.05. **(B)** Distribution of total scores by LLM. Tukey’s *post hoc* test following ANOVA revealed significant differences in total scores among models with mean differences of 1.09 (95% CI [0.249, 1.931], *p* = 0.007) for CHATGPT vs. BING, 1.87 (95% CI [1.029, 2.711], *p* < 0.001) for GEMINI vs. BING, and 0.78 (95% CI [−0.061, 1.621], *p* = 0.075) for GEMINI vs. CHATGPT. Adjusted *p*-values were used, with significance set at *p* < 0.05. **(C)** Distribution of the sum of total scores for each question on a per model basis.

Total scores for each question are shown in Figure 2A. The B12 Deficiency case, and SLE case were found to have significantly lower means scores compared against the total mean score of 11.4, suggesting the LLM output was of lower quality in these cases. In contrast, the Insulinoma case and SARS-ARDS case both had statistically higher scores, suggesting the LLM output was of higher subjective quality, and the models were more adept at these cases.

The cases used, and LLM output are provided in Supplementary material S1, and the observers scoring is provided in Supplementary material S2. The mean total scores per model were Bing 10.4, ChatGPT 11.5 and Gemini 12.3. The distribution of scores by model is shown in Figure 2B. The analysis of total scores among different models show significant differences. Specifically, the mean total score was 1.09 points higher for ChatGPT output compared to Bing (95% CI [0.249, 1.931], *p* = 0.007), and 1.87 points higher for Gemini compared to Bing (95% CI [1.029, 2.711], *p* < 0.001). However, the difference between Gemini and ChatGPT was not statistically significant (mean difference = 0.78, 95% CI [−0.061, 1.621], *p* = 0.075). These findings highlight distinct performance characteristics among the models, with Gemini showing the highest mean total score.

The performance of each model on each question is shown in Figure 2C. The total scores representing the sum of each observer’s score was highly variable across questions and models.

The analysis of average scores using the two-way consistency model demonstrated strong agreement among raters. The intraclass correlation coefficient (ICC) was calculated to be 0.892 (95% CI, 0.823–0.941), indicating a high level of consistency in scoring across the 30 subjects rated by 10 raters [*F*(29,261) = 9.23, *p* = 3.76e-26]. This finding supports the reliability of the scoring rubric.

## 4 Discussion

This report used readily available, consumer facing models and complex cases with sufficient red herrings and distractors so as to challenge a physicians. As a whole, the models performed best on relatively straightforward cases (SARS-ARDS and Insulinoma where there were few differentials), and performed least well in the most complex cases of B12 deficiency and SLE, which were the most undifferentiated presentations. This suggests a strength of LLM models may be in “rare” diseases such as insulinomas which, while rare, have distinct features, as opposed to vaguer, multisystem diseases such as SLE. The Bing model, while competent in easier cases, exhibited limitations in correctly diagnosing challenging cases. Furthermore, Bing is limited to a finite number of questions per day, impacting on its reliability and utility in a real-world environment.

We found that ChatGPT and Gemini both outperformed Bing, a finding that has been consistent when tested in a range of clinical scenarios including haematology cases, physiology cases vignettes, surgical decision making and dentistry (Dhanvijay et al., 2023; Giannakopoulos et al., 2023; Kumari et al., 2023; Lim et al., 2023; Gomez-Cabello et al., 2024; Lee et al., 2024a,b).

Our grading tool was easily understandable and quick to deploy, and demonstrated high interobserver reliability suggesting it may be of use to other researchers when assessing the diagnostic output of LLM models.

Limitations include the number of cases used which may impact generalisability, as well as assessing answers in a non-clinical environment, rather than assessing the outputs utility in clinical work setting in real time. Additionally, we restricted our assessment to English language output limiting generalisability to other languages.

The use of LLMS in clinical diagnostics presents significant ethical and data security challenges. Patient confidentiality is at risk when using such models. While it is possible to anonymise input to protect patient identity, it is entirely conceivable that identifiable data could be inadvertently inputted, or with enough contextual data (location, time, identity of user) the identity of a patient could be compromised. The models tested are not appropriate for real patient data due to the lack of data security.

Further, relying on LLMs to solve diagnostic cases raises questions around accountability in the event of errors, and without existing regulatory and ethical frameworks to support users, such tools may not be ready for formal integration into care pathways. However, as we have shown, the standard, consumer facing models available via their websites perform well, and the temptation to deploy such models remains – highlighting the urgency of the required frameworks.

Thus the exact role of such tools in a clinican’s work remains uncertain, and the need for oversight, as well as potential deskilling of staff (in particular junior staff) due to potential for overreliance in challenging cases which could impede learning.

Future work should systemically assess a greater number of cases to assess broader generalisability outside of internal medicine. These cases were selected from challenging scenarios with final diagnoses presented at the conclusion of the case which facilitated a comparison of output to a “ground truth.” An important translational step for future work will be to compare models in a clinical setting, with pragmatic analysis of applicability to real world cases assessed in real time, and where there is ambiguity as to the “true” diagnosis.

This research underscores the variation in model performance in clinical vignettes and highlights the importance of considering diagnostic model performance in diverse clinical scenarios. The findings suggest that model effectiveness varies based on the complexity of presented cases, and here we provide the community with a tool to help assess this output.

## Data availability statement

The raw data supporting the conclusions of this article will be made available by the authors, without undue reservation.

## Author contributions

MK: Data curation, Investigation, Methodology, Writing – original draft, Writing – review & editing. EO’S: Conceptualization, Formal analysis, Methodology, Supervision, Writing – original draft, Writing – review & editing.
