# Supplementary material for: A comparison of the diagnostic ability of large language models in challenging clinical cases
Source: Front Artif Intell. 2024 Aug 5;7:1379297. doi: 10.3389/frai.2024.1379297 (PMC11330891; doi:10.3389/frai.2024.1379297)
Supplement: Supplementary file 2 [file Data_Sheet_2.docx]

Case 1

A 15-Year-Old Girl with Abdominal Pain and an Ovarian Mass^1^

Correct diagnosis: Juvenile granulosa-cell tumour

Appropriate differential diagnosis: Ruptured ovarian cyst, epithelial cell tumours (e.g. mucinous cyst adenomas), germ cell tumours (e.g. cystic teratomas), sex cord stromal rumours (e.g. granulosa-cell tumours).

**CHATGPT_1**

Based on the presented information, the provisional diagnosis for this 15-year-old girl would be right ovarian torsion. Ovarian torsion occurs when the ovary twists on its vascular pedicle, leading to compromised blood flow to the ovary. This can result in significant abdominal pain.

The patient's history of sharp and crampy lower abdominal pain, which initially started in the midline and then shifted to the right side, is consistent with the presentation of ovarian torsion. The examination findings of mild tenderness on the right side of the lower abdomen and the presence of a complex, thick-walled cyst in the right ovary on ultrasound further support this diagnosis.

It's important to note that other potential causes of lower abdominal pain should also be considered and ruled out through further evaluation. Surgical consultation and imaging (such as CT or MRI) may be necessary to confirm the diagnosis and assess the degree of ovarian torsion.

**GEMINI_1**

The provisional diagnosis for a 15-year-old girl with abdominal pain and a complex, thick-walled cyst in the right ovary is ovarian cyst.

Ovarian cysts are common in women of all ages, but they are especially common in adolescents. Most ovarian cysts are benign and go away on their own without treatment. However, some ovarian cysts can be large or cause pain, and they may need to be removed surgically.

The symptoms of an ovarian cyst can vary depending on the size and type of cyst. Some women may have no symptoms at all, while others may experience pain, bloating, pressure in the pelvis, or irregular menstrual periods.

The most common type of ovarian cyst in adolescents is a functional cyst. Functional cysts form during the menstrual cycle and usually go away on their own within a few months.

Other types of ovarian cysts that can occur in adolescents include:

Endometriomas: These cysts form when endometrial tissue, the tissue that lines the uterus, grows outside of the uterus.

Dermoid cysts: These cysts contain a variety of tissues, including hair, teeth, and skin.

Mucinous cystadenomas: These cysts are filled with a thick, mucus-like fluid.

The diagnosis of an ovarian cyst is usually made based on a physical exam and imaging studies, such as an ultrasound or MRI.

The treatment for an ovarian cyst depends on the size and type of cyst. Most functional cysts do not require treatment and will go away on their own. However, larger cysts or cysts that cause pain or other symptoms may need to be removed surgically.

In this case, the patient's abdominal pain and the presence of a complex, thick-walled cyst on ultrasound suggest that she may have a functional ovarian cyst or another type of ovarian cyst, such as an endometrioma or dermoid cyst. Further evaluation with a gynecologist will be needed to confirm the diagnosis and determine the best course of treatment.

**BING_1**

Based on the information provided, the provisional diagnosis for the 15-year-old girl with abdominal pain is **a complex, thick-walled cyst in the right ovary**. [The presence of multiple follicular cysts in the left ovary and a small amount of free fluid in the pelvis also suggest an ovarian pathology^1^](https://www.aafp.org/pubs/afp/issues/2016/0515/p830.html).

[The differential diagnosis for acute abdominal pain in female adolescents includes pelvic inflammatory disease, pregnancy, ruptured ovarian cysts, or ovarian torsion^1^](https://www.aafp.org/pubs/afp/issues/2016/0515/p830.html). The patient’s negative urine test for human chorionic gonadotropin rules out pregnancy as a cause of her symptoms. [The absence of fever, bilious vomiting, bloody diarrhea, absent bowel sounds, voluntary guarding, rigidity, and rebound tenderness makes appendicitis less likely^1^](https://www.aafp.org/pubs/afp/issues/2016/0515/p830.html).

[Initial laboratory tests include complete blood count, erythrocyte sedimentation rate or C-reactive protein, urinalysis, and a pregnancy test^1^](https://www.aafp.org/pubs/afp/issues/2016/0515/p830.html). The patient’s white-cell count was elevated and hemoglobin level was low. However, blood levels of electrolytes, glucose, and lactate dehydrogenase were normal, as were the results of kidney-function and liver-function tests. [The urinalysis was normal^1^](https://www.aafp.org/pubs/afp/issues/2016/0515/p830.html).

Transabdominal ultrasonography with Doppler analysis revealed a complex, thick-walled cyst in the right ovary. There were multiple follicular cysts in the left ovary. Doppler flow was normal in both ovaries. There was a small amount of free fluid in the pelvis. [The appendix was not visualized^1^](https://www.aafp.org/pubs/afp/issues/2016/0515/p830.html).

Please note that this is a provisional diagnosis based on the information provided and further testing may be required to confirm the diagnosis.

Case 2

A 20-Year-Old Man with Shortness of Breath and Proteinuria^2^

Correct diagnosis: SLE (optional: with membranous lupus nephritis)

Appropriate differential diagnosis:

- Infective endocarditis
- Poststreptococcal glomerulonephritis
- Iga nephropathy or henoch–schönlein purpura nephritis
- Anca-associated vasculitis
- Anti-gbm disease

**CHATGPT_2**

Based on the patient's symptoms and history, the potential differential diagnoses could include:

1. Pulmonary embolism: The presence of emboli in both pulmonary arteries on CT angiography and the patient's symptoms of hemoptysis, shortness of breath, and chest pain are suggestive of pulmonary embolism.

2. Pneumonia: The initial diagnosis and treatment with doxycycline for presumed pneumonia might indicate that the patient had a higher risk for developing pneumonia, especially as the symptoms of cough, productive sputum, and fever persisted.

3. Pleural effusion: The presence of pleural effusion on imaging studies, specifically larger on the left side, suggests the possibility of infectious or inflammatory processes involving the lungs.

4. Asthma exacerbation: The patient's history of asthma and the presence of cough, shortness of breath, and wheezing on examination raise the possibility of an asthma exacerbation, especially considering his recent psychiatric hospitalization and stress.

Additional considerations for the differential diagnosis may include:

5. Chest trauma-related complications: The patient's history of chest trauma, including pain and subsequent nausea and vomiting, might have contributed to the development of complications such as rib fractures, lung contusion, or even pneumothorax.

6. Vasculitis: The presence of finger discoloration associated with temperature changes might suggest an underlying vasculitis, which can involve the lungs and lead to symptoms such as cough, hemoptysis, and shortness of breath.

7. Pulmonary infarction: The wedge-shaped opacities seen on CT, consistent with infarction, raise the possibility of pulmonary embolism or other vascular occlusion events causing infarction.

Further diagnostic tests, including blood cultures and additional imaging studies, might be necessary to narrow down the differential diagnosis and establish a definitive diagnosis.

**GEMINI_2**

The differential diagnosis for hemoptysis and hypoxemia in a 20-year-old man with bipolar disorder includes:

• Pulmonary embolism (most likely)

• Pneumonia

• Bronchitis

• Bronchiectasis

• Tuberculosis

• Lung cancer

• Pulmonary vasculitis

• Goodpasture syndrome

• Congestive heart failure

• Mitral stenosis

• Pulmonary hypertension

• Cystic fibrosis

• Bronchial foreign body

• Drug-induced pulmonary injury

The patient's clinical presentation is most consistent with pulmonary embolism. He has a history of a recent chest injury, which is a major risk factor for pulmonary embolism. He also has evidence of pulmonary infarction on CT angiography. Additionally, his hypoxemia and respiratory distress are consistent with pulmonary embolism.

However, it is important to consider other possible diagnoses, such as pneumonia and bronchiectasis, especially in a patient with a history of asthma. These conditions can also cause hemoptysis and hypoxemia.

The patient's bipolar disorder is not directly related to his hemoptysis and hypoxemia. However, some of his medications, such as quetiapine and paliperidone, can cause pulmonary side effects, such as pneumonia and bronchospasm.

Additional diagnostic tests that may be helpful include:

• Bronchoscopy to visualize the airways and look for evidence of bleeding or infection

• Sputum culture to identify any bacteria or fungi that may be causing pneumonia

• Antinuclear antibody (ANA) and anti-neutrophil cytoplasmic antibody (ANCA) tests to look for evidence of vasculitis

• Kidney function tests to look for evidence of kidney involvement in Goodpasture syndrome

• Echocardiogram to assess the heart function and look for evidence of mitral stenosis or pulmonary hypertension

• Pulmonary function tests to assess lung function and look for evidence of cystic fibrosis

Once a diagnosis has been established, the patient can be treated accordingly. Pulmonary embolism is typically treated with anticoagulants, such as warfarin or rivaroxaban. Pneumonia is treated with antibiotics. Bronchitis is typically treated with supportive care, such as rest, fluids, and over-the-counter cough medications. Bronchiectasis is treated with antibiotics and other therapies to help clear mucus from the lungs. Tuberculosis is treated with a long course of antibiotics. Lung cancer is treated with surgery, chemotherapy, and/or radiation therapy. Pulmonary vasculitis is treated with corticosteroids and other immunosuppressive medications. Goodpasture syndrome is treated with plasmapheresis and immunosuppressive medications. Congestive heart failure is treated with medications to improve heart function and reduce fluid buildup in the lungs. Mitral stenosis is treated with surgery or balloon valvuloplasty to open the narrowed valve. Pulmonary hypertension is treated with medications to lower blood pressure in the lungs. Cystic fibrosis is treated with a combination of medications, airway clearance techniques, and nutritional support. Bronchial foreign body is removed with bronchoscopy. Drug-induced pulmonary injury is treated by stopping the offending medication and providing supportive care.

**BING_2**

Based on the information provided, it appears that the patient has a history of bipolar disorder and presented with hemoptysis and hypoxemia. Six weeks before the current presentation, the patient was admitted under psychiatry for treatment of racing thoughts, generalized hyperactivity, and difficulty sleeping. During the admission, the patient was involved in an altercation and was struck on the left side of the chest. Five weeks before the current presentation, the patient was diagnosed with a pulmonary contusion. Ten days before the current presentation, the patient was evaluated in the emergency department and was diagnosed with pneumonia. A course of doxycycline was started for the treatment of presumed pneumonia, and the patient was discharged home. Four days before the current presentation, the course of doxycycline was completed. During the next several days, the cough increased in severity and became productive of yellow sputum with dark-red streaks, and shortness of breath developed; nausea also increased in severity. One day before the current presentation, the patient was evaluated again in the emergency department of another hospital. On evaluation, the patient appeared ill and had difficulty breathing. His temperature was 37.5°C, the blood pressure 99/50 mm Hg, the heart rate 127 beats per minute, the respiratory rate 31 breaths per minute, and the oxygen saturation 95% on room air 123.

Based on the information provided, the patient has a history of bipolar disorder and presented with hemoptysis and hypoxemia. The hemoglobin level was 8.7 g per deciliter and the albumin level 1.6 g per deciliter. Urinalysis revealed 2+ blood and 3+ protein. Differential diagnosis for the patient’s symptoms may include:

• Pneumonia: The patient was diagnosed with pneumonia 10 days before the current presentation 1.

• Pulmonary contusion: The patient was diagnosed with a pulmonary contusion 5 weeks before the current presentation 1.

• Pulmonary embolism: Pulmonary embolism can cause hemoptysis and hypoxemia 2.

• Lung cancer: Lung cancer can cause hemoptysis and hypoxemia 2.

• Tuberculosis: Tuberculosis can cause hemoptysis and hypoxemia 2.

• Bronchiectasis: Bronchiectasis can cause hemoptysis and hypoxemia 2.

• Acute respiratory distress syndrome (ARDS): ARDS can cause hypoxemia 3.

• Acute lung injury (ALI): ALI can cause hypoxemia 3.

• Pulmonary edema: Pulmonary edema can cause hypoxemia 3.

Please note that this is not an exhaustive list of differential diagnoses, and further diagnostic tests may be required to confirm the diagnosis.

CASE 3

A 49-Year-Old Man with Hypoglycemia^3^

Correct diagnosis: Insulinoma.

Acceptable differential dx: Systemic illness leading to hypoglycaemia (e.g. malnourishment and chronic disease, Addison’s), ingestion of drugs ( insulin or oral hypoglycaemic agents , multiple others – beta blockers, ACEi ) or toxins (e.g alcohol)

**CHATGPT_3**

Based on the given clinical presentation and history, the differential diagnosis for the 49-year-old man with hypoglycemia could include:

1. Insulinoma: A rare pancreatic tumor that causes excessive production of insulin leading to hypoglycemia.
2. Non-islet cell tumor hypoglycemia (NICTH): Tumors other than insulinomas, such as mesenchymal tumors or hepatocellular carcinoma, that can produce insulin-like growth factors and lead to hypoglycemia.
3. Hypoglycemia due to medication: Some medications, such as insulin or oral hypoglycemic agents, can cause hypoglycemia.
4. Adrenal insufficiency: Insufficient production of cortisol by the adrenal glands can lead to hypoglycemia.
5. Insulin autoimmune syndrome: A condition characterized by the presence of autoantibodies to insulin, causing hypoglycemia.
6. Malabsorption or malnutrition: Inadequate nutrient intake or malabsorption disorders can result in hypoglycemia.

Further investigations, such as imaging studies and hormone measurements, may be necessary to confirm the diagnosis and differentiate between the different possibilities. It is crucial that the patient receives appropriate evaluation and management under the supervision of a healthcare professional.

Bottom of Form

**GEMINI_3**

The differential diagnosis for hypoglycemia in a 49-year-old man includes:

- Insulin-induced hypoglycemia: This is the most common cause of hypoglycemia, especially in people with diabetes mellitus. Insulin can be administered exogenously (e.g., by injection, pump, or inhalation) or endogenously (e.g., by an insulinoma).
- Non-insulin-induced hypoglycemia: This can be caused by a variety of factors, including:
  - Medications: Certain medications, such as sulfonylureas, meglitinides, thiazolidinediones, and alcohol, can cause hypoglycemia.
  - Hormonal disorders: Adrenal insufficiency, hypopituitarism, and growth hormone deficiency can all lead to hypoglycemia.
  - Other medical conditions: Severe liver disease, kidney disease, and sepsis can also cause hypoglycemia.
  - Genetic disorders: There are a number of genetic disorders that can cause hypoglycemia, such as insulinoma, nesidioblastosis, and glycogen storage diseases.
  - Factitious hypoglycemia: This is a rare condition in which a person intentionally induces hypoglycemia, for example by injecting insulin.

In the case of this 49-year-old man, the following findings are suggestive of insulin-induced hypoglycemia:

- He has a history of unintentional weight gain and intermittent morning dizziness, which are both symptoms of insulin-induced hypoglycemia.
- He was found obtunded and making nonpurposeful movements, which are signs of severe hypoglycemia.
- His blood glucose level was very low on presentation.
- He responded rapidly to intravenous dextrose.

However, it is important to note that there are other possible causes of hypoglycemia in this patient, such as a hormonal disorder or a genetic disorder. It is also important to consider the possibility of factitious hypoglycemia.

To further investigate the cause of the patient's hypoglycemia, the following tests could be considered:

- Fasting serum insulin and C-peptide levels
- Plasma glucose and insulin levels during a 72-hour fast
- Abdominal imaging (e.g., CT or MRI scan) to look for an insulinoma

Once the cause of the patient's hypoglycemia has been identified, appropriate treatment can be initiated.

**BING_3**

Based on the information provided, the differential diagnosis for this patient’s hypoglycemia could include the following possibilities:

1. [**Diabetes**: Known diabetics are at risk of hypoglycemia due to the use of hypoglycemic agents such as sulfonylureas or insulin](https://litfl.com/hypoglycemia/)[^1^](https://litfl.com/hypoglycemia/).
2. [**Decreased glucose delivery**: Missed meals or overnight fasting can lead to hypoglycemia^1^](https://litfl.com/hypoglycemia/).
3. [**Increased glucose utilization**: Exercise can increase glucose utilization and potentially cause hypoglycemia^1^](https://litfl.com/hypoglycemia/).
4. [**Reduced endogenous glucose production**: Alcohol ingestion can reduce endogenous glucose production and lead to hypoglycemia](https://litfl.com/hypoglycemia/)[^1^](https://litfl.com/hypoglycemia/).
5. [**Increased insulin sensitivity**: Weight loss or an increase in exercise can increase insulin sensitivity and potentially cause hypoglycemia](https://litfl.com/hypoglycemia/)[^1^](https://litfl.com/hypoglycemia/).
6. [**Reduced insulin clearance**: Renal failure can reduce insulin clearance and lead to hypoglycemia](https://litfl.com/hypoglycemia/)[^1^](https://litfl.com/hypoglycemia/).
7. [**Exogenous drugs**: Certain medications, such as insulin, oral hypoglycemics, ethanol intoxication, quinine, chloroquine, beta-blocker overdose, valproate overdose, salicylate overdose, and pentamidine, can cause hypoglycemia^1^](https://litfl.com/hypoglycemia/).
8. [**Pituitary insufficiency**: Pituitary insufficiency can lead to hypoglycemia](https://litfl.com/hypoglycemia/)[^1^](https://litfl.com/hypoglycemia/).
9. [**Post-prandial hypoglycemia**: Late dumping syndrome after gastric surgery can cause post-prandial hypoglycemia](https://litfl.com/hypoglycemia/)[^1^](https://litfl.com/hypoglycemia/).
10. [**Liver disease**: Hepatocellular cancer, hepatitis, and rare genetic defects can cause hypoglycemia](https://litfl.com/hypoglycemia/)[^1^](https://litfl.com/hypoglycemia/).
11. [**Islet cell tumors**: Insulinomas are islet cell tumors that produce excessive insulin and can cause hypoglycemia](https://litfl.com/hypoglycemia/)[^1^](https://litfl.com/hypoglycemia/).
12. [**Immune hypoglycemia**: Anti-insulin receptor antibodies in Hodgkin’s disease or anti-insulin antibodies that release insulin when insulin levels are relatively low can cause immune hypoglycemia](https://litfl.com/hypoglycemia/)[^1^](https://litfl.com/hypoglycemia/).
13. [**Infection**: Severe sepsis or malaria can cause hypoglycemia](https://litfl.com/hypoglycemia/)[^1^](https://litfl.com/hypoglycemia/).
14. [**Non-pancreatic neoplasms**: Fibromas, sarcomas, mesotheliomas, and small cell carcinomas that produce IGF-2; extensive metastases that overwhelm the body’s ability to produce glucose can cause non-pancreatic neoplasms](https://litfl.com/hypoglycemia/)[^1^](https://litfl.com/hypoglycemia/).
15. [**Nesidioblastosis or noninsulinoma pancreatogenous hypoglycemia (NIPH) syndrome**: Islet cell hyperplasia, which can be congenital or acquired (e.g., post-gastric surgery), can cause nesidioblastosis or NIPH syndrome](https://litfl.com/hypoglycemia/)[^1^](https://litfl.com/hypoglycemia/).
16. [**Starvation and malnutrition**: Starvation and malnutrition can lead to hypoglycemia^1^](https://litfl.com/hypoglycemia/).
17. [**Hypothyroidism**: Myxedema coma due to severe hypothyroidism can cause hypoglycemia^1^](https://litfl.com/hypoglycemia/).
18. [**Pseudohypoglycemia**: Delayed measurement of a sample in the presence of leukocytosis, thrombocytosis, or erythrocytosis can result in pseudohypoglycemia^1^](https://litfl.com/hypoglycemia/).

Please note that this is not an exhaustive list of all possible causes of hypoglycemia and further evaluation by a healthcare professional is necessary for an accurate diagnosis.

Case 4

Posterior Reversible Encephalopathy Syndrome^4^

Correct diagnosis: Posterior reversible encephalopathy syndrome (PRES) (non-essential: due to cyclosporine use)

Acceptable Differential diagnosis: Inflammatory cerebral amyloid angiopathy, Progressive multifocal leukoencephalopathy, posterior circulation infarct, sagittal sinus thrombosis, hypoxic-ischaemic encephalopathy, severe hypoglycaemia.

**ChATGPT_4:**

Based on the information provided, a provisional diagnosis for this 35-year-old man could be posterior reversible encephalopathy syndrome (PRES), also known as reversible posterior leukoencephalopathy syndrome (RPLS).

**GEMINI_4**

Based on the clinical presentation and MRI findings, my provisional diagnosis is posterior reversible encephalopathy syndrome (PRES) related to cyclosporine therapy.

PRES is a clinico-radiological syndrome characterized by neurological symptoms (such as headache, seizures, altered consciousness, and visual disturbances), and characteristic MRI findings of vasogenic edema in the posterior white matter and cerebral cortex. It is thought to be caused by a breakdown of the blood-brain barrier, resulting in leakage of fluid and protein into the brain.

Cyclosporine is an immunosuppressive drug that is used to treat a variety of conditions, including IgA nephropathy. However, it can also have a number of side effects, including PRES.

The MRI findings in this case are consistent with PRES, showing hyperintensities in the cortical and subcortical white matter in a holohemispheric watershed pattern. The fact that the patient started developing neurological symptoms two weeks after starting cyclosporine further supports the diagnosis of PRES related to cyclosporine therapy.

Other possible diagnoses in this case include:

- Eclampsia/preeclampsia
- Hypertensive encephalopathy
- Acute disseminated encephalomyelitis (ADEM)
- West Nile virus encephalitis
- Methanol ingestion

However, these diagnoses are less likely based on the patient's clinical presentation and MRI findings.

It is important to note that a definitive diagnosis of PRES can only be made by a neurologist or other qualified healthcare professional.

**BING_4**

[Based on the information you provided, the 35-year-old man with IgA nephropathy who presented to the emergency department with confusion, blurry vision, and seizures might be experiencing **cyclosporine-induced encephalopathy**^1^](https://bmcnephrol.biomedcentral.com/articles/10.1186/s12882-017-0467-z). [Cyclosporine is an immunosuppressive medication used to treat IgA nephropathy and prevent organ rejection in transplant patients](https://bmcnephrol.biomedcentral.com/articles/10.1186/s12882-017-0467-z)[^1^](https://bmcnephrol.biomedcentral.com/articles/10.1186/s12882-017-0467-z)[^2^](https://nephcure.org/livingwithkidneydisease/treatment-options/cyclosporine/). [The patient’s symptoms, including confusion, blurry vision, and seizures, along with the presence of vasogenic edema on MRI, are consistent with cyclosporine-induced neurotoxicity^1^](https://bmcnephrol.biomedcentral.com/articles/10.1186/s12882-017-0467-z). It is important to note that this is a serious condition that requires immediate medical attention. I recommend consulting a healthcare professional for further evaluation and management.

Please keep in mind that I am an AI language model and cannot provide personal medical advice. It is always best to consult a qualified healthcare professional for accurate diagnosis and appropriate treatment options.

Case 5

Renal Cryptococcosis ^5^

Correct diagnosis: Renal Cryptococcosis

Acceptable differential diagnosis: other infective – e.g. ideally fungal infections coccidioidomycosis, histoplasmosis, tuberculosis

# ChatGPT_5

Based on the provided information, the diagnosis in this case is likely disseminated cryptococcosis. This is supported by the finding of abundant yeast cells with narrow-based budding and a thick, mucinous outer capsule in the glomeruli and tubulointerstitial compartment on renal biopsy. The mucicarmine stain identifying microorganism capsules and yeast forms on electron microscopy further supports the diagnosis of cryptococcosis. Furthermore, the patient's positive HIV status and low CD4+ count are consistent with an increased risk for opportunistic infections, such as cryptococcosis.

**GEMINI _5**

The diagnosis is **cryptococcal kidney disease**, a serious fungal infection that can occur in people with HIV/AIDS.

Cryptococcus neoformans is a fungus that is found in soil and bird droppings. It is a common cause of meningitis in people with HIV/AIDS, but it can also infect other organs, including the kidneys.

The most common symptoms of cryptococcal kidney disease are fever, chills, and flank pain. Some people may also experience nausea, vomiting, and loss of appetite. In severe cases, cryptococcal kidney disease can lead to kidney failure.

The diagnosis of cryptococcal kidney disease is made based on a combination of clinical findings, laboratory tests, and imaging studies. A blood test can detect the presence of cryptococcal antigen, and a urine test can detect the presence of cryptococcal organisms. An imaging study, such as a computed tomography (CT) scan or magnetic resonance imaging (MRI) scan, can be used to assess the severity of the kidney infection.

Treatment for cryptococcal kidney disease typically involves antifungal medications. In some cases, dialysis may be necessary to support kidney function until the infection is resolved.

The prognosis for cryptococcal kidney disease depends on the severity of the infection and the patient's overall health. With early diagnosis and treatment, most people with cryptococcal kidney disease can make a full recovery. However, the infection can be fatal in people with severe kidney damage or other underlying medical conditions.

**BING_5**

[Based on the information provided, the diagnosis for this 46-year-old man is **disseminated histoplasmosis**](https://symptomchecker.webmd.com/multiple-symptoms?symptoms=diarrhea%7Cweight-loss-%28unintentional%29&symptomids=72%7C257&locations=24%7C66)[^1^](https://symptomchecker.webmd.com/multiple-symptoms?symptoms=diarrhea%7Cweight-loss-%28unintentional%29&symptomids=72%7C257&locations=24%7C66). Disseminated histoplasmosis is a systemic fungal infection caused by the dimorphic fungus Histoplasma capsulatum. It is characterized by a wide range of clinical manifestations, including fever, weight loss, hepatosplenomegaly, and mucocutaneous lesions. [The infection can disseminate to various organs, including the lungs, liver, spleen, bone marrow, and gastrointestinal tract](https://symptomchecker.webmd.com/multiple-symptoms?symptoms=diarrhea%7Cweight-loss-%28unintentional%29&symptomids=72%7C257&locations=24%7C66)[^1^](https://symptomchecker.webmd.com/multiple-symptoms?symptoms=diarrhea%7Cweight-loss-%28unintentional%29&symptomids=72%7C257&locations=24%7C66).

In this case, the patient’s clinical presentation is consistent with disseminated histoplasmosis. [The symptoms of diarrhea, nausea, abdominal pain, and weakness along with weight loss are common manifestations of the disease^1^](https://symptomchecker.webmd.com/multiple-symptoms?symptoms=diarrhea%7Cweight-loss-%28unintentional%29&symptomids=72%7C257&locations=24%7C66). The positive enzyme-linked immunosorbent assay (ELISA) for human immunodeficiency virus (HIV) indicates that the patient is immunocompromised. [HIV infection is a major risk factor for disseminated histoplasmosis^1^](https://symptomchecker.webmd.com/multiple-symptoms?symptoms=diarrhea%7Cweight-loss-%28unintentional%29&symptomids=72%7C257&locations=24%7C66).

[Laboratory studies revealed a low CD4+ count (2 cells per cubic millimeter), which further supports the diagnosis of disseminated histoplasmosis in an immunocompromised individual^1^](https://symptomchecker.webmd.com/multiple-symptoms?symptoms=diarrhea%7Cweight-loss-%28unintentional%29&symptomids=72%7C257&locations=24%7C66). [The renal biopsy findings of abundant yeast cells with narrow-based budding and a thick, mucinous outer capsule are consistent with Histoplasma capsulatum infection^1^](https://symptomchecker.webmd.com/multiple-symptoms?symptoms=diarrhea%7Cweight-loss-%28unintentional%29&symptomids=72%7C257&locations=24%7C66).

[Disseminated histoplasmosis is a serious condition that requires prompt treatment with antifungal medications such as amphotericin B or itraconazole^1^](https://symptomchecker.webmd.com/multiple-symptoms?symptoms=diarrhea%7Cweight-loss-%28unintentional%29&symptomids=72%7C257&locations=24%7C66). I recommend consulting an infectious disease specialist for further evaluation and management.

Case 6

Necrotizing anterior scleritis^6^

Correct diagnosis: Necrotizing anterior scleritis

Appropriate differentials: episcleritis ( essential – key differential ), herpes zoster, scleromalacia perforans

**CHATGPT_6**

Based on the provided information, the most likely diagnosis for this patient is scleromalacia perforans. Scleromalacia perforans is a rare complication of rheumatoid arthritis characterized by thinning and necrosis of the sclera, which can result in the exposure of underlying choroid. It typically presents with pain, redness, and inflammation in the affected eye. Laboratory findings of an elevated rheumatoid factor and increased erythrocyte sedimentation rate are consistent with the patient's history of seropositive erosive rheumatoid arthritis. The negative results on testing for antineutrophil cytoplasmic antibodies help to exclude granulomatosis with polyangiitis as a potential cause. Further confirmation can be obtained through a thorough ophthalmologic examination, which may include a scleral biopsy. Treatment involves managing the underlying rheumatoid arthritis and may include immunosuppressive therapies.

**GEMINI_6**

Based on the clinical presentation and laboratory findings, the most likely diagnosis is necrotizing scleritis. Necrotizing scleritis is a severe form of scleritis, which is an inflammation of the sclera, or the white part of the eye. It is characterized by the destruction of the scleral tissue, which can lead to thinning and even perforation of the eye.

Necrotizing scleritis is more common in people with autoimmune disorders, such as rheumatoid arthritis. It can also be caused by infections or other inflammatory conditions.

The patient in this case has a history of seropositive erosive rheumatoid arthritis, which is a severe form of rheumatoid arthritis. She also stopped her immunosuppressive treatment four years ago, which may have made her more susceptible to developing necrotizing scleritis.

The clinical presentation is also consistent with necrotizing scleritis. The patient has pain, hyperemia, inflammation, and marked scleral thinning in her right eye. The laboratory findings also support the diagnosis, as the patient has an elevated level of rheumatoid factor and an increased erythrocyte sedimentation rate.

Other possible diagnoses in this case include:

- Peripheral ulcerative keratitis (PUK) is a type of corneal ulcer that can occur in people with rheumatoid arthritis. However, PUK is typically unilateral and does not involve scleral thinning.
- Mooren's ulcer is another type of corneal ulcer that can occur in people with rheumatoid arthritis. However, Mooren's ulcer typically progresses slowly and does not involve scleral thinning.
- Scleromalacia perforans is a rare type of scleritis that is characterized by thinning and perforation of the sclera without inflammation. However, scleromalacia perforans is typically unilateral and does not involve hyperemia or inflammation.

Given the patient's history, clinical presentation, and laboratory findings, the most likely diagnosis is necrotizing scleritis.

Treatment

Treatment for necrotizing scleritis typically involves high-dose corticosteroids and immunosuppressive drugs. The goal of treatment is to suppress the inflammation and prevent further destruction of the sclera. In some cases, surgery may be necessary to repair the damage to the sclera.

Prognosis

The prognosis for necrotizing scleritis varies depending on the severity of the disease and the response to treatment. Early diagnosis and treatment are essential to prevent vision loss and perforation of the eye.

**BING_6**

[Based on the information provided, the diagnosis for the 73-year-old woman with a history of seropositive erosive rheumatoid arthritis and pain in her right eye is **scleromalacia perforans**](https://consultqd.clevelandclinic.org/investigation-of-persistent-knee-pain-and-swelling-leads-to-uncommon-diagnosis/)[^1^](https://consultqd.clevelandclinic.org/investigation-of-persistent-knee-pain-and-swelling-leads-to-uncommon-diagnosis/). [Scleromalacia perforans is a rare ocular manifestation of rheumatoid arthritis characterized by scleral thinning, inflammation, and exposure of the underlying choroid^1^](https://consultqd.clevelandclinic.org/investigation-of-persistent-knee-pain-and-swelling-leads-to-uncommon-diagnosis/). [It is typically associated with long-standing, severe, and uncontrolled rheumatoid arthritis](https://consultqd.clevelandclinic.org/investigation-of-persistent-knee-pain-and-swelling-leads-to-uncommon-diagnosis/)[^1^](https://consultqd.clevelandclinic.org/investigation-of-persistent-knee-pain-and-swelling-leads-to-uncommon-diagnosis/). [The patient’s history of seropositive erosive rheumatoid arthritis, elevated rheumatoid factor, increased erythrocyte sedimentation rate, and negative results on testing for antineutrophil cytoplasmic antibodies are consistent with this diagnosis](https://consultqd.clevelandclinic.org/investigation-of-persistent-knee-pain-and-swelling-leads-to-uncommon-diagnosis/)[^1^](https://consultqd.clevelandclinic.org/investigation-of-persistent-knee-pain-and-swelling-leads-to-uncommon-diagnosis/).

Case 7

B12 Deficiency^7^

Correct diagnosis: B12 Deficiency

Appropriate differentials: primary psychosis, secondary psychosis: substance use, endocrine (thyroid or Wilsons), delirium, cerebral infection, neurodegenerative disorder (e.g. Huntington’s), inflammatory (SLE), seizures

**CHATGPT_7**

Hepatic Encephalopathy: The patient's elevated ammonia levels, abnormal liver function tests (elevated alanine aminotransferase), and cognitive impairment could suggest hepatic encephalopathy, which can present with neurological symptoms such as confusion, delusions, and disorganized behavior.

Differential Diagnoses:

Acute Liver Failure: The abnormal liver enzymes, low hemoglobin, and low platelet count could indicate acute liver failure, which can manifest with hepatic encephalopathy and neurological symptoms.

Metabolic Encephalopathy: The patient's abnormal electrolyte levels and hematological abnormalities could be indicative of metabolic disturbances contributing to encephalopathy and neurological symptoms.

Drug-Induced Hepatotoxicity: Despite the negative urine and serum toxicology screenings, drug-induced hepatotoxicity leading to hepatic encephalopathy cannot be entirely ruled out, especially if the patient had been exposed to hepatotoxic substances not included in routine toxicology screening.

Vitamin B1 Deficiency (Wernicke's Encephalopathy): The low hemoglobin and low hematocrit levels could suggest nutritional deficiencies, such as vitamin B1 deficiency, which can present with neurological symptoms and delusional thinking.

Given these findings, further investigation is warranted, including a detailed review of the patient's medication history, a thorough assessment for signs of liver dysfunction, consideration of nutritional deficiencies, and additional imaging studies to evaluate the liver and brain. The patient's management should involve addressing the underlying cause, supportive care, and monitoring for potential complications.

**GEMINI_7**

Final Diagnosis (Undetermined at this point)

Given the ongoing evaluation, a definitive diagnosis cannot be made yet. More information from the additional tests mentioned is needed.

The laboratory results provide some valuable insights that can help refine the differential diagnosis:

Electrolytes:

Potassium is slightly low (3.3 mmol/liter), which can contribute to muscle weakness and tingling (paresthesia).

Liver Function Tests:

Mildly elevated alkaline phosphatase (ALP), Alanine aminotransferase (ALT), and Lactate dehydrogenase (LDH) suggest possible liver injury.

Kidney Function Tests:

Normal results for these tests suggest the kidneys are functioning normally.

Blood Cell Counts:

Microcytic anemia (low red blood cell count, hemoglobin, and hematocrit with high mean corpuscular volume) suggests iron deficiency or another cause.

Elevated eosinophils could indicate an allergic reaction or parasitic infection.

Other:

Elevated prolactin could be related to a pituitary gland issue or medications.

Elevated lactate could be a sign of tissue hypoxia (oxygen deprivation).

Revised Differential Diagnosis:

Psychiatric Disorder: Still a strong possibility, but the laboratory findings suggest investigating further medical causes for the patient's symptoms.

Metabolic Disorder:

Vitamin B12 deficiency: While some lab values aren't classic for B12 deficiency (normal mental status exam and neurologic exam on initial presentation), the low potassium and microcytic anemia warrant further investigation.

Hypokalemia (low potassium) can be caused by various factors, including malnutrition, medications, and some psychiatric disorders.

Liver Disease: The mildly elevated liver enzymes could be related to various conditions, including:

Alcoholic liver disease (less likely given the reported history of no alcohol use).

Fatty liver disease (more likely considering the patient's weight).

Medication side effects.

Autoimmune Disease: Elevated prolactin and some other lab findings could point towards an autoimmune disorder, but further investigation is needed.

Infectious Disease: Eosinophilia could indicate a parasitic infection, but other symptoms and tests are needed to confirm.

Next Steps:

Further evaluation for Vitamin B12 deficiency, including levels of methylmalonic acid and homocysteine.

Investigation into the cause of the mildly elevated liver enzymes.

Additional tests might be needed depending on the specific suspected diagnosis.

BING_7

Certainly! Let’s consider the information provided and generate a diagnosis and differential diagnosis:

## Diagnosis:

- **Delusional Disorder**:
  - The patient’s disheveled appearance, pressured speech, and delusions of persecution (fear of unwanted sleep studies or being gassed) are suggestive of a primary psychotic disorder.
  - Delusional thinking is a hallmark feature of delusional disorder.
  - Further psychiatric evaluation is needed to confirm this diagnosis.

## Differential Diagnosis:

1. **Schizophrenia**:
   - Schizophrenia presents with positive symptoms (hallucinations, delusions) and negative symptoms (social withdrawal, flattened affect).
   - Consider if the patient has other features consistent with schizophrenia.
2. **Substance-Induced Psychosis**:
   - Although urine toxicology screening was negative, consider less common substances or designer drugs.
   - Reevaluate the possibility of substance-induced psychosis.
3. **Metabolic Encephalopathy**:
   - Abnormal laboratory findings (elevated CK, LDH, low hemoglobin, hematocrit) may suggest an underlying metabolic disturbance.
   - Explore electrolyte imbalances, vitamin deficiencies, or thyroid dysfunction.
4. **Other Organic Causes**:
   - Neuroimaging (MRI) showed mild mucosal thickening in the paranasal sinus but no acute intracranial abnormalities.
   - Consider other organic causes (e.g., infections, autoimmune conditions) that could affect cognition.
5. **Educational Background and Intelligence**:
   - The patient’s unconventional educational experiences may be relevant.
   - Could her educational background contribute to her symptoms?
6. **Collateral Information Limitations**:
   - Lack of family or friends for collateral information complicates the assessment.
   - Efforts to obtain historical information are crucial.

## Next Steps:

- Collaborate with a multidisciplinary team.
- Monitor laboratory trends.
- Continue psychiatric evaluation and initiate appropriate treatment.
- Consider additional neuroimaging or specialized tests.
- Explore resources for the patient’s well-being and safety.

Case 8

HNf1B-related disease^8^

Correct diagnosis: HNF1B related disease

Acceptable differential diagnosis: CAKUT, ADPKD, nephronophthisis

**CHATGPT_8**

Based on the provided information, the diagnosis and differential diagnosis could be:

Diagnosis:

The patient presents with multiple kidney cysts, pancreatic hypoplasia, and a history of diabetes mellitus. Given these findings, the diagnosis is likely:

Juvenile Nephronophthisis (NPHP): This is a rare genetic disorder characterized by the development of multiple cysts in the kidneys, often leading to renal failure in adolescence or early adulthood. NPHP can also present with extrarenal manifestations such as pancreatic anomalies and diabetes mellitus.

Differential Diagnoses:

Other conditions to consider in the differential diagnosis include:

Polycystic Kidney Disease (PKD): Although less likely due to the patient's age and absence of a family history of PKD, this condition presents with the development of multiple cysts in the kidneys and can also involve extrarenal manifestations.

Medullary Cystic Kidney Disease (MCKD): Another rare genetic disorder characterized by cyst formation in the renal medulla, which may present with similar imaging findings. However, MCKD typically presents later in life than NPHP.

Diabetes Mellitus: Although the patient has a history of diabetes mellitus, the presence of kidney cysts and pancreatic anomalies suggests that the diabetes may be secondary to the underlying renal disorder rather than a primary diagnosis.

Congenital Anomalies of the Kidney and Urinary Tract (CAKUT): Certain CAKUT, such as renal dysplasia or multicystic dysplastic kidney, can present with cystic changes in the kidneys. However, the association with pancreatic anomalies and diabetes mellitus is less common in these conditions.

Further evaluation, including genetic testing for NPHP-related genes and renal biopsy if necessary, would help confirm the diagnosis. Additionally, close monitoring of renal function and management of diabetes mellitus are essential components of the patient's care plan.

**GEMINI_8**

More information on the results of the diagnostic test performed in the nephrology clinic is needed for a definitive diagnosis.

Most likely diagnosis: Autosomal Dominant Polycystic Kidney Disease (ADPKD): This is a genetic disorder causing multiple cysts in both kidneys. However, the patient's age at presentation (19 years old) is atypical for ADPKD, which usually presents in adulthood.

Differential Diagnosis

Based on the information presented so far, the following conditions are on the differential diagnosis list for the kidney cysts:

Autosomal Recessive Polycystic Kidney Disease (ARPKD): Another genetic disorder with multiple kidney cysts. It typically presents in infancy or early childhood, which isn't the case here.

Nephronophthisis (Nephronic Cystic Disease): This is a genetic disorder with progressive kidney failure and multiple kidney cysts.

Tuberous Sclerosis Complex (TSC): This is a genetic disorder with various manifestations, including kidney cysts.

Medullary Cystic Kidney Disease (MCKD): This is a group of inherited disorders causing cysts in the inner part (medulla) of the kidneys.

Acquired Cystic Kidney Disease: This can be caused by long-term exposure to nephrotoxins (toxins affecting the kidneys).

Other Considerations:

The patient's diabetes could be related to a genetic syndrome that also causes kidney cysts.

The mildly elevated liver enzymes and absent pancreas on imaging could indicate another underlying condition.

The next steps likely involve:

Analyzing the results of the diagnostic test performed in the nephrology clinic.

Depending on the test results, further genetic testing might be needed.

**BING_8**

The patient’s clinical presentation, laboratory findings, and imaging results suggest a complex interplay of kidney cysts and diabetes. Let’s explore the possibilities:

Renal Cysts and Diabetes Syndrome (RCAD):

RCAD, also known as MODY 5, is characterized by:

Cystic renal disease: The presence of multiple bilateral simple kidney cysts.

Early-onset familial non-autoimmune diabetes: Typically occurs before age 25.

HNF1B gene mutation: Alterations in the HNF1B gene lead to both renal cysts and impaired insulin production.

Pancreatic atrophy: The pancreas may be smaller than expected.

[Liver dysfunction and genital tract anomalies can also be features of RCAD1](https://www.medicalnewstoday.com/articles/renal-cysts-and-diabetes-syndrome)[2](https://www.mendelian.co/diseases/renal-cysts-and-diabetes-syndrome-rcad).

Simple Renal Cysts:

Simple kidney cysts are fluid-filled pouches originating from the kidney surface.

They are usually harmless unless they increase in size, rupture, or exert pressure on adjacent organs.

The patient’s bilateral simple kidney cysts, ranging from 2 to 18 mm, may be incidental findings.

[No specific treatment is necessary unless complications arise3](https://microsoftstart.msn.com/en-au/health/ask-professionals/in-expert-answers-on-cyst/in-cyst?questionid=v4l1wtag&type=condition&source=healthpaa_conditionqna).

Other Considerations:

The elevated liver enzymes (AST, ALT, ALP, GGT) observed in the gastroenterology clinic warrant further evaluation.

The patient’s history of elevated liver enzymes 1 year before the current presentation may be relevant.

Additional investigations (e.g., liver function tests, imaging) are needed to assess liver function and rule out other liver diseases.

Given the patient’s age and clinical features, RCAD remains a strong possibility. However, close follow-up and multidisciplinary management are crucial to address both renal and metabolic aspects of this complex condition.

Case 9

SARs-ARDS^9^

Correct Diagnosis: SARS-Cov2- ARDS

Acceptable differential diagnosis: infectious pneumonia, pulmonary embolisms, interstitial pneumonia e.g. cryptogenic organizing pneumonia and acute eosinophilic pneumonia. aspiration pneumonitis, cardiogenic pulmonary oedema, vasculitis.

**CHATGPT_9**

In light of the laboratory data provided, the diagnosis and differential diagnosis for the 81-year-old man with fever, cough, and shortness of breath can be revised:

Diagnosis:

Given the new laboratory data, which show significant abnormalities consistent with severe illness and multi-organ involvement, the diagnosis is likely:

Severe COVID-19 Pneumonia with Multi-organ Dysfunction: The patient's symptoms, along with laboratory findings such as elevated lactic acid, abnormal liver function tests (elevated ALT, AST, alkaline phosphatase, total bilirubin), elevated inflammatory markers (CRP, ESR), elevated ferritin, and evidence of coagulopathy (elevated d-dimer, prolonged PT/INR, elevated fibrinogen), suggest severe COVID-19 pneumonia with associated multi-organ dysfunction syndrome (MODS).

Differential Diagnoses:

Sepsis: The clinical presentation and laboratory findings overlap with severe sepsis or septic shock, which can also present with fever, respiratory symptoms, elevated inflammatory markers (CRP, ESR), and evidence of multi-organ dysfunction. However, the ongoing pandemic of COVID-19 and the characteristic radiographic findings make severe COVID-19 pneumonia more likely.

Pulmonary Embolism (PE) with Sepsis: Although less likely given the characteristic radiographic findings of COVID-19 pneumonia, the combination of severe illness and elevated d-dimer could raise concern for concurrent pulmonary embolism. However, in the context of the pandemic and typical laboratory findings of severe COVID-19, this diagnosis is less likely.

Acute Respiratory Distress Syndrome (ARDS): Severe COVID-19 pneumonia can progress to ARDS, characterized by bilateral pulmonary infiltrates, hypoxemia, and respiratory distress. The patient's presentation is consistent with this complication.

Acute Decompensated Heart Failure: Given the patient's history of hypertension and chest pain, acute decompensated heart failure could be considered. However, the laboratory findings, including elevated inflammatory markers and coagulation abnormalities, are more indicative of severe COVID-19 pneumonia with associated complications.

Given the severity of the patient's illness and the pandemic context, prompt supportive management for COVID-19 pneumonia, including oxygen therapy, fluid resuscitation, and potential consideration for systemic corticosteroids and anticoagulation, is warranted. Close monitoring for complications such as acute respiratory distress syndrome (ARDS), sepsis, and multi-organ dysfunction is essential.

**GEMINI_9**

Working Diagnosis: COVID-19 Pneumonia with Possible Complications

While a definitive diagnosis can't be confirmed yet without the results of the diagnostic test, the information provided suggests COVID-19 pneumonia as the most likely working diagnosis. Here's why:

Symptoms at presentation (fever, cough, and shortness of breath) align with COVID-19.

The ongoing pandemic strengthens the possibility of COVID-19 infection.

Chest X-ray findings (bilateral patchy airspace opacities) are consistent with COVID-19 pneumonia.

Possible Complications:

The chest pain and shortness of breath could indicate:

Pleural effusion: Fluid accumulation between the lung and chest wall, which can cause pleuritic pain.

Pulmonary embolism (PE): A blood clot lodged in a lung artery, which can cause pleuritic pain and shortness of breath.

Underlying lung disease: The patient's past medical history suggests pre-existing lung disease that could worsen with COVID-19 infection.

The performed diagnostic test result is crucial for confirmation:

A positive SARS-CoV-2 RNA test from the nasopharyngeal swab would confirm COVID-19 infection.

Depending on the suspected complications (PE or pleural effusion), further tests like CT scan or chest ultrasound might have been performed.

It's important to note that this is a working diagnosis based on the limited information provided. A qualified healthcare professional can provide a definitive diagnosis after a complete evaluation.

**BING_9**

This **81-year-old man** presents with **fever**, **cough**, and **shortness of breath** during the Covid-19 pandemic. Let’s consider the differential diagnosis based on the provided information:

1. **Covid-19 (SARS-CoV-2 Infection)**:
   - Given the pandemic context, Covid-19 is a significant consideration.
   - Symptoms include fever, cough, and dyspnea.
   - The **bilateral patchy airspace opacities** seen on chest radiograph are consistent with viral pneumonia.
   - Confirmatory testing with **SARS-CoV-2 PCR** or antigen test is essential.
2. **Chronic Obstructive Pulmonary Disease (COPD)**:
   - The patient’s history of **hypertension** and **chronic lung disease** (with lung tissue hardening) raises suspicion.
   - COPD can cause chronic cough, shortness of breath, and chest pain.
   - Inspiratory crackles may be due to underlying lung disease.
3. **Pulmonary Embolism (PE)**:
   - PE can present with sudden onset of dyspnea, chest pain (especially pleuritic), and hypoxemia.
   - Risk factors include age, immobility, and hypertension.
   - Consider **CT pulmonary angiography** to evaluate for PE.
4. **Heart Failure (HF)**:
   - HF can cause dyspnea, especially at rest or with exertion.
   - Elevated blood pressure and tachycardia are relevant.
   - **Bilateral crackles** may be due to pulmonary congestion.
5. **Other Infectious Causes**:
   - Besides Covid-19, consider other respiratory infections (e.g., **pneumonia**, **influenza**).
   - Blood cultures and sputum analysis may help identify the causative agent.
6. **Bronchiectasis**:
   - The patient’s history of lung disease and **bronchiectasis** may contribute to symptoms.
   - Bronchiectasis can cause chronic cough and recurrent infections.
7. **Other Cardiac Causes**:
   - **Myocardial infarction (MI)** can present with chest pain and dyspnea.
   - **Aortic dissection** can cause chest pain and hypoxemia.

Given the patient’s age, history, and radiographic findings, Covid-19 remains a top consideration. However, further investigations (e.g., SARS-CoV-2 testing, CT angiography, echocardiogram) are necessary to confirm the diagnosis. Urgent management includes oxygen supplementation and close monitoring in the hospital setting.

Case 10

sepsis^10^

Correct diagnosis: Meningococcal purpura fulminans or meningococcal sepsis

Appropriate Differential diagnosis: Skin necrosis, Thrombocytopenia purpura, cryoglobulinaemic vasculitis, catastrophic antiphospholipid syndrome, infectious purpura fulminans

**CHATGPT_10**

Based on the presented case, a potential diagnosis for the 19-year-old man could be Toxic Shock Syndrome (TSS), specifically Staphylococcal Toxic Shock Syndrome (TSS) or Streptococcal Toxic Shock Syndrome. TSS is a rare but potentially life-threatening condition characterized by fever, hypotension, rash, multiorgan involvement, and desquamation (peeling of the skin) following a toxin-mediated response to certain bacterial infections.

Differential Diagnoses:

Septic Shock: This is a severe condition resulting from an overwhelming infection leading to systemic inflammation and organ dysfunction. It shares some clinical features with TSS, including fever, hypotension, and multiorgan involvement.

Meningococcal Septicemia: Neisseria meningitidis infection can lead to a fulminant form of sepsis known as meningococcemia, which can present with a rash, fever, and shock. Neck stiffness and headache can also be present.

Acute Intoxication: Some toxic substances, such as certain drugs or chemicals, can cause symptoms similar to those seen in TSS, including nausea, vomiting, abdominal pain, and altered mental status.

Acute Pancreatitis: Severe pancreatitis can present with abdominal pain, nausea, vomiting, and shock-like symptoms. Elevated lipase levels may be present on laboratory testing.

Acute Viral Syndrome: Certain viral infections, such as severe influenza or viral hemorrhagic fevers, can present with fever, myalgias, rash, and multiorgan involvement.

Anaphylaxis: In severe cases, anaphylactic reactions to food or medications can present with shock, respiratory distress, rash, and gastrointestinal symptoms.

Given the patient's history of consuming food before symptom onset and the rapid progression to shock and multiorgan failure, toxic shock syndrome secondary to a bacterial toxin is a leading consideration. However, further diagnostic evaluation, including blood cultures, toxin assays, and other specific tests, would be needed to confirm the diagnosis and differentiate it from other potential causes.

**GEMINI_10**

Working Diagnosis: Sepsis with Multisystem Organ Failure (Likely Meningococcal Sepsis)

This case presentation suggests sepsis with multisystem organ failure as the most likely working diagnosis. Here's why:

The rapid onset of symptoms like fever, chills, nausea, vomiting, muscle aches, rash, and altered mental status is characteristic of sepsis.

The development of shock (low blood pressure despite fluids), respiratory failure (need for mechanical ventilation), and kidney dysfunction further supports sepsis with multisystem organ failure.

The petechial rash described is a classic sign of meningococcal sepsis, a potentially life-threatening bacterial infection.

Additional factors supporting meningococcal sepsis:

Young age (19 years old) is a risk factor for meningococcal sepsis.

The recent travel to a crowded place (music venue) increases the chance of exposure to the bacteria.

The result of the diagnostic test performed 7 hours after admission is crucial for confirmation:

A positive blood culture or PCR test for Neisseria meningitidis, the bacteria causing meningococcal sepsis, would confirm the diagnosis.

It's important to note that this is a working diagnosis based on the limited information provided. A definitive diagnosis can be confirmed by the results of the diagnostic test and a complete evaluation by a healthcare professional.

**BING_10**

The 19-year-old man in this case presents with shock, multiple organ failure, and a purpuric rash. Let’s consider the differential diagnosis based on the provided information:

Meningococcal Sepsis (Neisseria meningitidis):

Meningococcal sepsis can cause a rapidly progressive illness with shock, purpura, and multi-organ failure.

The patient’s purpuric rash, neck stiffness, and headache are suggestive of meningococcal infection.

However, other typical features such as petechiae on the skin and Kernig sign (resistance to knee extension when the hip is flexed) are not mentioned.

A lumbar puncture would be necessary to confirm or rule out meningitis.

Empiric treatment with broad-spectrum antibiotics (e.g., ceftriaxone) should be initiated promptly.

Purpura Fulminans:

Purpura fulminans is a severe form of disseminated intravascular coagulation (DIC) characterized by widespread purpura, skin necrosis, and multi-organ failure.

The patient’s purpuric rash, cyanosis, and multiple organ failure fit this diagnosis.

It can be triggered by various infections, including meningococcal, Streptococcus pneumoniae, and Capnocytophaga.

Laboratory tests (e.g., coagulation studies) would help confirm DIC.

Toxic Shock Syndrome (TSS):

TSS can result from toxin-producing bacteria (e.g., Staphylococcus aureus or Streptococcus pyogenes).

The patient’s shock, rash, and multi-organ failure are consistent with TSS.

TSS can be associated with tampon use, skin infections, or post-surgical wounds.

Treatment involves supportive care, antibiotics, and removal of the source (e.g., tampon).

Other Infectious Causes:

Consider other bacterial, viral, or fungal infections that can lead to sepsis and multi-organ failure.

Blood cultures, urine analysis, and nasopharyngeal swabs are essential for identifying the causative agent.

Drug Reaction or Toxin Exposure:

The patient’s symptoms began after consuming leftovers from a restaurant meal.

Consider food poisoning, toxin exposure, or an allergic reaction.

The yellow-green vomitus may be relevant.

Other Causes of Shock and Multi-organ Failure:

Non-infectious etiologies such as adrenal crisis, hemorrhagic shock, or metabolic disorders should also be considered.

Given the severity of the presentation, the patient requires urgent resuscitation, broad-spectrum antibiotics, and close monitoring in the intensive care unit. Further investigations, including blood cultures and imaging, are crucial for narrowing down the diagnosis. Consultation with specialists (e.g., infectious disease, hematology) is recommended.

Please note that this is a preliminary differential, and additional information and test results would be needed for a definitive diagnosis. The patient’s condition warrants immediate attention and management.
